# My Brain Needs a Break: Kindergarteners’ Willpower Theories Are Related to Behavioral Self-Regulation

**DOI:** 10.3389/fpsyg.2020.601724

**Published:** 2020-12-16

**Authors:** Miriam Compagnoni, Vanda Sieber, Veronika Job

**Affiliations:** ^1^Institute of Education, Faculty of Arts and Social Sciences, University of Zurich, Zurich, Switzerland; ^2^Faculty of Psychology, Technische Universität Dresden, Dresden, Germany

**Keywords:** implicit theories about willpower, self-regulation, motivation, kindergarten, goal-orientation, self-control, willpower, meta-cognition

## Abstract

Is the way that kindergarteners view their willpower – as a limited or as a non-limited resource – related to their motivation and behavioral self-regulation? This study is the first to examine the structure of beliefs about willpower in relation to behavioral self-regulation by interviewing 147 kindergarteners (52% girls) aged 5 to 7 years (*M* = 6.47, *SD* = 0.39). A new instrument was developed to assess implicit theories about willpower for this specific age group. Results indicated that kindergarteners who think of their willpower as a non-limited resource showed better behavioral self-regulation than children who adopted a more limited theory, even when controlling for age and gender. This relation was especially pronounced in low achieving children. Mediation and moderation analyses showed that this relation was partly mediated through the children’s willingness to invest effort to reach a learning goal. Findings suggest that fostering metacognitive beliefs in children, such as the belief that willpower is a non-limited resource, may increase behavioral self-regulation for successful adjustment to the demands of kindergarten and school.

## Introduction

Childhood behavioral self-regulation is the capacity to focus and maintain attention on tasks and follow instructions to consciously regulate the self in line with goals, including the capacity to inhibit unwanted thoughts, feelings, or impulses; it is a key predictor for successful learning and adjustment to school and life (McClelland et al., 2007; Moffitt et al., 2013; Baumeister et al., 2018). Research suggests that successful self-regulation and goal striving depend on people’s beliefs – or implicit theories – about the nature of willpower (Job et al., 2010, 2015b). These implicit theories capture whether people think of their willpower as a limited resource that becomes depleted easily and needs to be replenished by taking a break, eating, or resting (limited willpower theory) or as something that is more stable and even becomes energized by previous strenuous self-control tasks (non-limited willpower theory). Previous studies with adults have shown that people who believe that willpower is non-limited (vs. limited) exhibit better self-control in tasks in the laboratory (Job et al., 2010; Miller et al., 2012) and better self-regulation in everyday life (Bernecker et al., 2015; Job et al., 2015b). Even if the beneficial effects of a non-limited theory in adults are well-understood, it is not known whether young children already have ideas about willpower and whether those ideas affect their self-regulation. To investigate these research gaps we conducted a study in kindergartens in Switzerland, where children attend a two-year program in their local public schools starting at age 4 or 5. In line with previous research on willpower theories (Job et al., 2010, 2015b), we propose that non-limited theories are especially beneficial when demands on self-regulation are high, which is often the case for academic underachievers. We further assume that the relation between willpower theories and behavioral self-regulation is mediated by children’s willingness to exert effort to learn something (i.e., learning goal orientation; Compagnoni et al., 2019).

In summary, four main questions guided the present study: (1) Can willpower theories be reliably assessed in kindergarteners? (2) Are kindergarteners’ willpower theories related to their behavioral self-regulation? (3) Is this association stronger in children with low academic ability levels? and (4) Is the relation between willpower theories and behavioral self-regulation mediated by children’s learning goal orientation?

### Self-Regulation in Children

There is a consensus that self-regulation is a key predictor of success in school and life, and childhood years are seen as a sensitive period for development of self-regulation (Cameron Ponitz et al., 2008; Kubesch, 2014; Blair and Raver, 2015; McClelland et al., 2018). However, there is no common definition of childhood self-regulation and the different research directions lack integration (for a discussion see Panadero, 2017; McClelland et al., 2018). Early childhood research has emphasized basic skills that underlie self-regulated action, such as focusing and maintaining attention on tasks, working memory, and inhibitory control (Sektnan et al., 2010). Some researchers refer to these self-regulatory skills as executive functions and focus on cognitive processes (Blair and Razza, 2007; Rothlisberger et al., 2010; Moriguchi and Hiraki, 2013). However, other researchers term this set of skills “behavioral self-regulation” and subsequently adopt a broader view of the regulation of behavior (McClelland et al., 2007; Suchodoletz et al., 2014). Research on self-control (or willpower), in turn, focuses more on one specific skill – inhibitory control – and defines willpower as the capacity to override unwanted thoughts, feelings, or impulses to align the self with long-term goals (Mischel et al., 2011; Baumeister et al., 2018). But Mischel et al. (2011) also state that willpower “requires that individuals encode only information from the environment that is relevant, keeping wanted information active in working memory and suppressing unwanted information and selecting desired responses while withholding responses that are not optimal” (p. 254), which refers to a very similar set of underlying basic skills. The conceptual clutter and overlap in constructs related to early behavioral self-regulation can partly be explained by different measurement methods (McClelland et al., 2019) but also by the high correlations between the basic components of behavioral self-regulation (Willoughby et al., 2011; Bull and Lee, 2014). They often cannot be distinguished in young children, seem to gradually differentiate with age, and subserve successful context-specific behavioral self-regulation and self-control (Hofmann et al., 2012).

Impaired behavioral self-regulation has been described as being accountable for many educational and societal issues, ranging from learning difficulties, job underperformance, behavioral problems at school, and violence to obesity (Duckworth and Seligman, 2005; Baumeister et al., 2007; Moffitt et al., 2013). Mixed results emerge regarding the strength of the relation with academic achievement level, depending on cultural context, measurement method, or academic domain. However, overall, previous research makes a strong case for the importance of behavioral self-regulation in the educational context (McClelland et al., 2007; Gestsdottir et al., 2014). As children who improved their self-regulation – independent from their initial level – showed better outcomes in adulthood (Moffitt et al., 2011), the question of how to promote self-regulation is occupying teachers and researchers around the world. The assumption that self-regulation can be trained analogously to a muscle (Baumeister et al., 2007) led to several successful programs for developing self-regulation through repeated practice in challenging situations (Diamond and Lee, 2011; Rothlisberger et al., 2012; Rybanska et al., 2018). Whereas the research presented above focuses on innate prerequisites and the training of self-regulation, other research suggests that successful self-regulation also depends on people’s beliefs – or implicit theories – about the nature of their abilities (e.g., intelligence, Dweck and Leggett, 1988; or willpower, Job et al., 2010).

Over the last decades, Dweck and colleagues (Dweck and Leggett, 1988; Mueller and Dweck, 1998; Gunderson et al., 2013; Haimovitz et al., 2019) have shown that children develop implicit theories about their abilities as being either fixed (fixed mindset) or capable of growing (growth mindset) based on experiences such as praise and feedback for success and failure. Children with a growth mindset show better behavioral self-regulation, as they embrace challenges as learning opportunities to grow and improve their abilities (Molden and Dweck, 2006; Burnette et al., 2013; Compagnoni et al., 2019). In contrast, children with a fixed mindset, who view their abilities as stable traits, are concerned about their performance (Perry et al., 2019) and show poorer behavioral self-regulation (Dweck and Leggett, 1988; Dweck, 2017). As a consequence, they are more likely to avoid challenging tasks, see effort as a sign of weakness, and adopt poor self-regulation strategies (for a meta-analytic review see Burnette et al., 2013). Researchers have found that people not only hold such implicit beliefs about their abilities but also about other characteristics, such as individual traits (e.g., Chiu et al., 1997). Most important for research on self-regulation is the finding that people also have specific implicit theories about their willpower (Mukhopadhyay and Johar, 2005; Job et al., 2010). When people think of their willpower as a limited resource that becomes easily depleted and needs to be replenished by taking a break, eating, or relaxing (limited theory), their self-control capacity becomes impaired when they face self-regulatory challenges (Job et al., 2010). In contrast, people who believe that their willpower is something that is more stable and that even becomes energized by previous strenuous self-control exertion (non-limited theory) are better able to sustain their self-control at high levels when they encounter difficulties.

### Willpower Theories as Predictor of Behavioral Self-Regulation

Previous studies with adults have shown that people who believe that willpower is non-limited (vs. limited) showed better self-regulation in everyday life, such as procrastinating less and following a healthier diet (Job et al., 2015b), more progress on personal goals (Bernecker et al., 2015), greater sustained learning (Miller et al., 2012), higher academic achievement in university students facing high demands (Job et al., 2015b), and improved self-efficacy regarding upcoming tasks (Bernecker et al., 2015). The relation between willpower theories and self-control holds in adults when beliefs about willpower are measured as an individual difference and also when they are manipulated experimentally (Job et al., 2010).

As studies with experimentally induced as well as measured willpower theories in adults show that a non-limited willpower theory is associated with various aspects of self-regulation, we expect a relation with behavioral self-regulation in children. Especially in Swiss kindergartens, which offer children the possibility to choose tasks, task level, and task engagement during free play, demands of self-regulation are high (Hauser, 2013). Thus, willpower theories are expected to have a strong impact on behavioral self-regulation. We assume that a child with a non-limited willpower theory might persist in the face of difficulties, inhibit the impulse to give up or take a break, and therefore train self-regulation, improve self-efficacy, and choose more challenging tasks in the future. Children who struggle with a task and, in contrast, think of their willpower as something limited will be motivated to rest and replenish their resources when experiencing a task as exhausting. They might not persist and therefore their self-efficacy regarding upcoming tasks will be diminished. As a consequence, they might choose fewer challenging tasks in the future. Since only one study has examined the correlates of willpower theories in young children (Haimovitz et al., 2019), there is a paucity of research examining the role that willpower theories play in explaining early self-regulation. Haimovitz et al. (2019) found that children with experimentally induced non-limited theories (through a model in a storybook who experiences exerting willpower as energizing) showed more self-control strategies in the face of temptation, spent more time on the strategies, and showed longer delay of gratification than the group of children with a experimentally induced limited theory. The results imply that diverse behavioral self-regulation may develop not just as a set of skills learned through repeated practice on challenging tasks, as examined in past research (Diamond and Lee, 2011). A general approach to willpower that encourages children’s generation and use of self-control strategies by shaping their willpower theories might be effective too. Although this is promising, further research still needs to establish if children in kindergarten already have naturally occurring beliefs about their willpower and if they are related to a set of skills such as behavioral self-regulation, which subsequently leads to better academic outcomes.

According to Haimovitz et al. (2019), in early childhood children may not yet have well-formed beliefs about their willpower. They found that in children aged 4–5 years, the exposure to a storybook model that experiences exerting willpower as energizing leads to improved behavioral self-regulation. But as they did not measure children’s beliefs about willpower before and after the manipulation, they could not distinguish whether an existing willpower theory was modified or a new idea about willpower was implanted. Based on some researchers, it seems even plausible that most children generally have a non-limited theory, as they are usually overoptimistic and think that with enough effort they can master almost anything (Hasselhorn, 2005). Given the lack of research on willpower theories in children, it remains unclear whether kindergarteners already have well-formed willpower theories, if they vary between children, if they can be measured reliably, and if they are already related to behavioral self-regulation as early as kindergarten age.

Taken together, we assume that willpower is not just a skill but is already rooted in a mental model about the nature of willpower that might encourage children to seek (or discourage them from seeking) effective strategies to meet high self-regulatory demands that can help them execute high behavioral self-regulation (Yeager and Dweck, 2012).

### Academic Ability Level as Moderator

Educational research on diverse motivational beliefs has demonstrated that a growth mindset (Paunesku et al., 2015) or high self-concepts (Compagnoni and Losenno, 2020) are especially beneficial for academic underachievers. This finding has been explained by the notion that motivational beliefs play an important role especially in challenging situations when behavior must be actively self-regulated and when automatisms and routines can no longer be maintained. Similarly, research on implicit theories about willpower documented that endorsing a non-limited theory is mostly beneficial when a person is facing high self-regulatory demands (Bernecker et al., 2015; Job et al., 2015b). Since everyday life in kindergarten is associated with greater challenges for children with lower academic ability levels, especially in open learning environments (Helmke, 2009; Hauser, 2013), a greater impact of willpower theories in children with low academic abilities can be expected. As children with low academic abilities might generally experience tasks as more challenging, those with a limited theory might often find themselves in situations where they readily give up when they struggle with a task. As a consequence, they spend less time training their self-regulation than children with low academic ability levels and a non-limited theory. Additionally, children who are perceived as having low academic achievement levels by teachers might be allowed to take a break after strenuous tasks when they ask for it (or even be encouraged to do so), which might undermine their self-regulation. A non-limited theory might prevent low achieving children from prematurely asking teachers for a break or switch tasks when faced with difficulties. To date, there are no studies examining the moderating effect of children’s academic ability level on the association between willpower theories and behavioral self-regulation.

Research on adults’ implicit theories about willpower, however, suggests that when demands are high, a non-limited willpower theory promotes self-regulation directly, by keeping self-efficacy high (Chow et al., 2015) and by preventing a premature shift of motivation away from a task and toward rest and recovery (Job et al., 2015a). Including academic ability as a moderator will allow us to determine if the positive relation between willpower theories and behavioral self-regulation is especially pronounced in low achievers.

### Goal Orientation as Mediator

We propose that non-limited willpower theories additionally affect self-regulation indirectly through the children’s higher willingness to exert effort in order to learn and increase their competencies by embracing challenging tasks. This orientation toward mastery motivates children to approach opportunities to train behavioral self-regulation and is based on the conviction that learning requires time and effort. It has been termed “learning goal orientation” or “mastery approach goal orientation” (Dweck and Leggett, 1988; Perry et al., 2017). A learning goal orientation is positively related to persistence in the face of failure and enhanced motivation toward challenging tasks (Dweck and Leggett, 1988) and seems to be a hallmark for training self-regulation (Perry et al., 2017). Past findings suggest that a learning goal orientation plays a pivotal role in linking implicit theories about intelligence and self-regulation processes (Smiley and Dweck, 1994; Compagnoni et al., 2019). Growth mindset interventions have found that children who were led to adopt a learning goal orientation changed their view of challenges (Burnette et al., 2013).

In the present study, we suggest that children with a non-limited willpower theory might be more prone to adopt a high learning goal orientation due to their enhanced willingness to invest effort. In turn, they should be more likely to choose more difficult tasks, which train their behavioral self-regulation. In contrast, children with a limited theory might choose easy tasks that they already master to not deplete their resources. For example, a kindergarten child with a learning goal orientation might choose to play a new difficult game with numbers over replaying a familiar game, even though it requires attention and persistence and success is not guaranteed. In contrast, a performance orientation is associated with engaging in easy tasks that one can master quickly with minimal effort (Bakadorova et al., 2020).

This study extends past literature on willpower theories in adults and takes up questions raised in Haimovitz et al. (2019) experimental study with preschoolers. We measured kindergarten children’s beliefs about willpower and looked into the relation between these beliefs and behavioral self-regulation. We hypothesized that kindergarteners already have varying beliefs about their willpower, which can be measured reliably when age appropriate measurement methods are applied. Further, we expected that the more kindergarteners view their willpower as non-limited, the better their behavioral self-regulation. In line with previous research on willpower theories in academic contexts, we proposed that non-limited theories are especially beneficial when demands on self-regulation are high, which is often the case for academic underachievers. We therefore hypothesized that the direct relation between willpower theories and behavioral self-regulation is more pronounced for children on lower academic ability levels. We further assumed that the relation of willpower theories and behavioral self-regulation is mediated by the children’s learning goal orientation.

## Materials and Methods

### Participants

The sample included 147 children at 19 kindergartens in urban and rural areas that reflect the demographic composition of the German-speaking part of Switzerland. Only children whose parents had given written informed consent participated. For six children there was no consent to participate because the teacher only provided information to the parents the day before the assessment; parents of five children did not consent for personal reasons (religion, parents in divorce); and two children did not give a reason. We received parents and children’s informed consent to participate for 91% of the children attending the kindergartens. The children were in their second year of kindergarten and had an age range of 65–86 months (*M* = 6.47 years, *SD* = 0.39 years). Approximately half of the sample (52%) consisted of girls, 72% of the children in the sample were of Swiss nationality, and 45% spoke Swiss German as their first language, 10% spoke Albanian, 3–7% spoke Serbian/Croatian, Turkish, German, Portuguese, English, German, Spanish, and Arabic, respectively, and the rest spoke other first languages. In Switzerland kindergartens are part of the public education system, and 95% of children attend a 2-year kindergarten program in their local public schools starting at age 4 or 5 (Swiss Conference of Cantonal Ministers of Education, 2017), resulting in a body of children with diverse economic status and ethnicities. All participants were following the official curriculum guidelines for kindergartens in Switzerland, where free play in an open learning environment is emphasized (Hauser, 2013).

### Procedure

Due to the short attention span in children of this age, the children were visited twice in their kindergartens with an interval of 2 to 4 weeks between the assessments. The questionnaires were each administered in the context of a larger battery of cognitive tasks in a session that lasted approximately 30 min. Given the kindergarten children’s lack of reading and writing skills, tests were administered through an individual interview procedure. Willpower theories and goal orientation were assessed at both visits with Versions A and B of the questionnaires (see Supplementary Material). Behavioral self-regulation was measured at the end of the second visit. Teachers completed an online questionnaire between the two visits. All children received a small gift for participating, and the class received a math game. Missings in the data were due to children who were absent at one of the two measurement points (*t*_1_: *n* = 4, *t*_2_: *n* = 5) or technical network failures.

### Measures

#### Behavioral Self-Regulation

Behavioral self-regulation, the ability to focus and maintain attention on a task and inhibit inappropriate actions, was measured with the Head-Toes-Knees-Shoulders (HTKS) task (Cameron Ponitz et al., 2008). We used the newer, more complex version of the HTKS that was developed for older children (see McClelland et al., 2014) to prevent ceiling effects (Gestsdottir et al., 2014). This direct observational measure taps into the three aspects of executive functions: inhibitory control, attention, and working memory. A single measure was used for behavioral self-regulation, as replicated findings of several studies in preschool-aged children found a lack of differentiation into distinct components (Bull and Lee, 2014; Clark et al., 2014). Children were asked to play a game in which their task was to do the opposite of what the experimenter said (e.g., “touch your head!” and then they had to touch their toes instead). The first 10 trials included two types of paired commands, for the next 10 trials two new paired commands were added, and for the last 10 trials, all four commands were re-paired into new commands combinations (e.g., “touch your shoulder!” and then they had to touch their toes instead). The 30 items were scored with 0 for an incorrect response, 1 for a self-corrected response, and 2 for a correct response (*M* = 41.70, *SD* = 10.36, Cronbach’s α = 0.89). Higher scores indicated higher levels of behavioral self-regulation.

#### Willpower Theories

To assess limited and non-limited willpower theories in kindergarteners, we developed a self-report method with age-appropriate items based on the measure used by Job et al. (2010) and inspired by the Berkeley Puppet Interview (Measelle et al., 1998). A researcher asked two identical puppets named “Mi” and “Mo” standardized questions (e.g., “Does your brain need many breaks during strenuous thinking?”), and the children listened to the puppets’ answers delivered on a touchscreen. One puppet expressed a limited theory (i.e., “Yes, whenever I have done something strenuous, my brain needs a break”) and the other a non-limited theory (i.e., “Not at all, my brain can think as long as it wants”). The children indicated on a 5-point semantic differential scale displayed on the touchscreen between the two puppets (1 = *limited theory*, 5 = *non-limited theory*) which of the puppets they could identify themselves with (“Are you more like Mi or more like Mo?”). As suggested by Marsh et al. (2002), a double binary response strategy was used to counter the tendency to select endpoints and neglect intermediate points: The identification with one puppet (by pressing a button) was always followed by a second probe (“Do you totally agree with this puppet, or do you agree only a little?”). Items from Job et al. (2010) were translated and adapted to the age group. In a pilot phase with 10 children using think aloud method, items were tested and adapted in several iterative loops resulting in six items (see Supplementary Material). Although we chose visually identical, gender neutral puppets, the puppets statements were randomized to ensure that childrens’ answers did not express sympathies for one puppet. Children completed the two questionnaires during the two visits. Version A and B were similar in content and were combined to create a single willpower theory score for each child. Confirmatory factor analyses (CFA) confirmed a one factor solution [χ2(9) = 12.696, *p* = 0.177, RMSEA = 0.055, *p-close* = 0.399, TLI = 0.949, CFI = 0.970, SRMT = 0.042]. A more restricted two factor solution [*χ2*(8) = 12.671, *p* = 0.124, RMSEA = 0.065, *p-*close = 0.307, TLI = 0.928, CFI = 0.962, SRMT = 0.042, Δχ*2* (1) = 0.025, *p* = 0.874] showed a slightly worse fit and revealed a correlation of 0.980 between version A and B, indicating the stability of the construct. The final willpower theory scale consisted of 6 items, *M* = 2.89, *SD* = 1.05, skew = 0.327 (0.206), kurtosis = −0.532 (0.410), Cronbach’s α = 0.71, with higher scores associated with a non-limited theory.

#### Learning Goal Orientation

Children’s learning goal orientation, their willingness to exert effort to learn something (Compagnoni et al., 2019), was assessed with 6 Items adapted from Gunderson et al. (2013)’s motivational framework measures. To capture learning goal orientation, the same procedure was used as for willpower theories. A researcher asked two puppets standardized questions, and the children listened to the puppets’ answers delivered on a touchscreen. One puppet expressed a stronger learning goal orientation (i.e., “I like to do difficult tasks to learn something”) than the other (i.e., “I like to do easy tasks to get it right”). The children indicated how much they could identify with one of the two puppets on a semantic differential scale with five points displayed on the touchscreen between the puppets. Items on learning goal orientation were mixed with items on willpower theories and assessed during the two visits. Version A (three items) and B (three items) were similar in content and were combined to create a single goal orientation score for each child. Higher scores are associated with a higher learning goal orientation. The learning goal orientation scale consisted of six items, *M* = 3.66, *SD* = 1.18, Cronbach’s α = 0.88.

#### Covariates

Gender, age, and academic ability level were assessed with an online questionnaire administered to the teachers. As no formal grades are given in kindergarten, we asked teachers to assess students’ academic abilities in mathematics and language. To increase the comparability and validity of the teachers’ global performance assessments, the teachers were given three examples each to integrate in their assessment of the mathematics domain (knowing numbers, calculate, count) and language domain (knowing letters, reading words, writing words). Teachers had to rate each child in their class on a 9-point semantic differential scale displayed by stick figures in a horizontal row. Lower values indicated a lower level of academic ability. The achievement measure used in this study therefore reflects a social reference standard, similar to grades (*M* = 6.21, *SD* = 2.04, Cronbach’s α = 0.84). The measure was part of a questionnaire assessing children’s’ ability self-concepts on the same scales (Cimeli et al., 2013).

### Data Analysis

Analyses were conducted using IBM SPSS Statistics 25.0. Based on *a priori* analyses with the G^∗^Power software package (Faul et al., 2009), for linear multiple regression analyses with up to five predictors we targeted a minimum sample size of 92 children to achieve a power of 0.80 (fixed model, R-squared deviation from zero, alpha level = 0.05, effect size *f*^2^ = 0.15). Since we allowed all children at the contacted schools to participate when they gave consent, the analyses were calculated with the complete sample of *N* = 147. To test whether willpower theories predicted behavioral self-regulation and whether this relationship was moderated by academic ability level, a three-stage hierarchical multiple regression was conducted with behavioral self-regulation as the dependent variable. Control variables were considered at stage one, willpower theories at stage two, academic ability level at stage three. Concerning the question as to whether the relation between willpower theories and behavioral self-regulation was mediated by children’s learning goal orientation, a simple mediation analysis was carried out. As we estimated our models with no *a priori* constraints on direct effects and the modeling of latent variables would reduce the power for our sample size, an OLS regression approach for estimating mediation models (Hayes, 2018) was chosen over a maximum likelihood based SEM program. Further OLS regression is more appropriate in small samples than SEM due to the *p*-values derived from t distributions. Both mediation and moderation analyses were conducted with a regression-based approach in SPSS using the macro PROCESS with bias corrected and accelerated bootstrap intervals estimates (Hayes, 2018). Bootstrapping as a non-parametric resampling procedure seemed reasonable to test the significance of a mediation effect, as it does not rely on the assumption of normality and is adequate for small sample sizes. Following the recommendations of Hayes (2018) regression analyses which include a product of predictors in the model are reported and interpreted based on unstandardized coefficients. In regression models without a product term as a predictor (Hayes, 2018, p. 313), standardized regression coefficients were generated and can be interpreted accordingly. As the children were clustered in kindergartens, we checked whether the application of a multilevel model would be necessary, although this would hardly be methodologically applicable for our small sample of 19 kindergartens. Small ICCs for all main variables between *ρ* = 0.002 for willpower theories and *ρ* = 0.111 for behavioral self-regulation with non-significant Wald z values indicated that there were no significant differences in willpower theories, goal orientation, age, academic ability ratings, or behavioral self-regulation across kindergartens.

## Results

Table 1 shows means, standard deviations, and range of willpower theories, goal orientation, behavioral self-regulation measures, academic ability level, and the covariates as well as zero-order rank correlations among all constructs. Kindergarteners’ willpower theories showed approximately the same medium correlation with teacher ratings of academic abilities (*r* = 0.226) and behavioral self-regulation (*r* = 0.219). As expected from previous research on the positive relation between self-regulation and academic ability level, results showed medium correlations of *r* = 0.363 between behavioral self-regulation assessed with the HTKS and academic ability level.

**TABLE 1:** Descriptives.

	*N*	Range	*M*	*SD*	SR	WT	GO	AAL	Gender	Age
Behavioral self-regulation (SR)	142	12–57	41.70	10.36	–	0.227**	0.223**	0.352**	–0.101	0.132
Willpower theories (WT)	138	1–5	2.89	1.05	0.219**	–	0.264**	0.246**	0.109	0.104
Learning goal orientation (GO)	138	1–5	3.66	1.18	0.227**	0.285**	–	0.288**	0.224**	0.136
Academic ability level (AAL)	142	1–9	6.21	2.04	0.363**	0.226**	0.269**	–	–0.010	0.165
Gender	147	1–2	1.48	0.50	–0.113	0.116	0.186*	–0.010	–	0.129
Age in months	143	65–86	77.21	4.67	0.118	0.176*	0.131	0.154	0.147	–

Non-parametrical Spearman correlations above the diagonal, parametrical Pearson correlation below the diagonal. Gender (girls = 1, boys = 2), *p < 0.05, **p < 0.01 (two-tailed).

### Structure of Willpower Theories

As we developed new items tailored to this age group to assess willpower theories, it is recommended that exploratory factor analysis (EFA) should precede CFA (Worthington and Whittaker, 2006). Both items to assess learning goal orientation and willpower refer to children’s motivational beliefs about dealing with challenges and were assessed with the same instrument. Therefore, to clarify the data structure, we conducted a principal axis component analysis (PAF) on the 12 items with oblique rotation, as we expected the factors to be moderately correlated. The Kaiser-Meyer-Olkin (KMO) measure verified the sampling adequacy to the analysis, KMO = 0.851. EFA yielded a 2-factor solution (43.14% EV), with a scree plot that justified two factors. Factor loadings after rotation showed reasonable item loadings from 0.416 to 0.814 on the two factors (Worthington and Whittaker, 2006). The two factors, learning goal orientation and willpower theories, showed a significant medium correlation of 0.302. To determine the theoretically assumed factor structure of the data, we applied CFA with MLM estimators that are robust to non-normality and skewed data, as is the case with goal orientation. A two-dimensional model with learning goal orientation and willpower theories computed as two related first-order latent factors immediately fit the data well [χ2 (53) = 50.108, *p* = 0.588; RMSEA = 0.000, 90% CI [0.000;0.049], TLI = 1.008, CFI = 1.000, SRMR = 0.052]. Given these findings, we constructed a willpower theories scale (Cronbach’s α = 0.71) and a learning goal orientation scale (Cronbach’s α = 0.88) for the two first-order factors.

### Relation of Willpower Theories With Behavioral Self-Regulation

Our second research question addressed the relation between willpower theories and behavioral self-regulation. Because empirical studies reported inconclusive or culturally different results regarding age and gender differences in behavioral self-regulation (Gestsdottir et al., 2014; Montroy et al., 2016; Yamamoto and Imai-Matsumura, 2017) with no difference or better behavioral self-regulation in girls and older children, gender and age were included as covariates. Results from the hierarchical regression model (Table 2) revealed in step 1 that gender and age explained 2% of the variance in behavioral self-regulation, with girls regulating better than boys and older children better than younger ones, but on a non-significant alpha level (*p* = 0.104). Introducing willpower theories in step 2 led to a significant change in *R*^2^ of additional 6% of explained variance in predicting behavioral self-regulation (*p* = 0.005) and established willpower theories as an important predictor of behavioral self-regulation (β = 0.243, *p* = 0.005). The more children thought of their willpower as non-limited, the better was their behavioral self-regulation. If academic ability level is included in step three, an additional 10% of the variance in behavioral self-regulation can be explained (*p* = 0.000). Regression coefficients in Table 2 show that willpower theories still contributed significantly to explain the variance in behavioral self-regulation (β = 0.175, *p* = 0.036).

**TABLE 2:** Hierarchical regression model for behavioral self-regulation.

Model		*B*	*SE B*	β	*p*	CI_95_ lower	CI_95_ upper	*R*^2^	*p*
1	*Constant*	23.207	14.696		0.117	–5.862	52.277		
	Age	0.297	0.192	0.134	0.124	–0.082	0.677		
	Gender	–3.045	1.807	–0.146	0.094	–6.619	0.528		
								0.019	0.104
2	*Constant*	23.730	14.315		0.100	–4.588	52.049		
	Age	0.208	0.190	0.094	0.275	–0.167	0.583		
	Gender	–3.509	1.767	–0.168	0.049	–7.005	–0.013		
	Willpower theories	2.429	0.851	0.243	0.005	0.754	4.114		
								0.069	0.006
3	*Constant*	20.977	13.614		0.126	–5.956	47.910		
	Age	0.133	0.181	0.060	0.463	–0.225	0.491		
	Gender	–3.301	1.679	–0.158	0.051	–6.623	0.021		
	Willpower theories	1.756	0.827	0.175	0.036	0.120	3.392		
	Academic ability level	1.641	0.420	0.319	0.000	0.809	2.472		
								0.161	0.000

*Model 1: *F*(2, 132) = 2.302, Δ*R*^2^ = 0.034, Δ*p* = 0.104; Model 2: *F*(3, 131) = 4.331, Δ*R*^2^ = 0.057, Δ*p* = 0.005; Model 3: *F*(4, 130) = 7.409, Δ*R*^2^ = 0.095, Δ*p* = 0.000.*

#### Moderation by Academic Ability Levels

**FIGURE 1:**
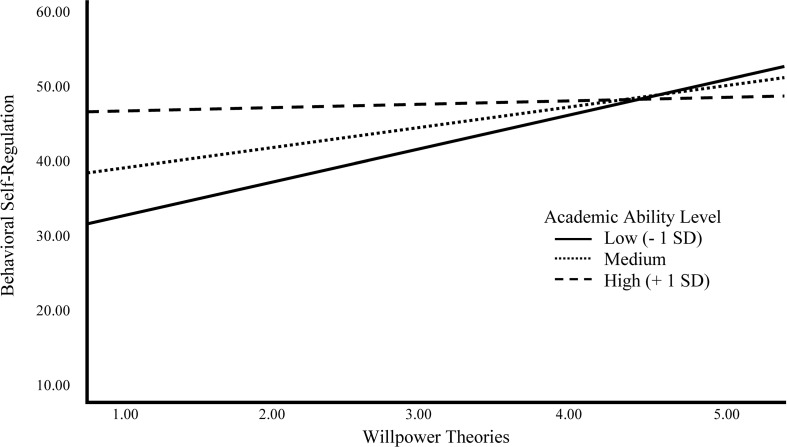
Graphical depiction of the moderation of the effect of willpower theories on behavioral self-regulation by academic ability level using *M* ∓ 1 *SD* to represent low, moderate and high values on the moderator.

Although the results showed that willpower theories are related to behavioral self-regulation, we hypothesized that the relation would be moderated by academic ability levels. Table 3 shows results of a regression analysis examining the moderation of willpower theories to behavioral self-regulation by academic ability level, controlling for age and gender using PROCESS (Hayes, 2018). Results of the moderation analysis showed that 23% of the variance in behavioral self-regulation could be explained by willpower theories, academic ability level, gender, and age. The relation between willpower theories and behavioral self-regulation was significantly moderated by academic ability level [*F*(1,129) = 7.801, *p* = 0.006], with an effect size of 5% increase in variance (Δ*R*^2^ = 0.046). A graphical depiction of the interaction revealed that behavioral self-regulation was especially low among children with low academic abilities which tended toward a limited willpower theory (see Figure 1). The analyses showed that the conditional direct effect of willpower theories on self-regulation was significant in children with low academic ability levels (*M*_–1*SD*_ = 4.149, *b* = 4.078, *SE* = 1.158, *p* = 0.001, 95% CI [1.787, 6.368]) and in children with moderate academic ability levels (*M* = 6.189, *b* = 2.014, *SE* = 0.811*, p* = 0.014, 95% CI [0.409, 3.619]). In contrast, willpower theories were not significantly related to behavioral self-regulation of children with a high academic ability level (*M*_+1*SD*_ = 8.229, *b* = −0.049, *SE* = 1.033, *p* = 0.962, 95% CI [−2.093, 1.995]). To ensure that the results of the interaction analysis were not caused by a statistical artifact due to low variance of willpower theories in children with high academic abilities, the sample was divided into three groups (low achievement level, *n* = 44, *M* = 2.63, *SD* = 0.94, medium achievement level, *n* = 47, *M* = 2.79, *SD* = 1.01; high achievement level, *n* = 47, *M* = 3.23, *SD* = 1.11) and compared regarding their variance. All three groups showed a range from a limited (1) to a non-limited theory (5) and no difference in variance (Levene’s test, *F* (2,135) = 0.801, *p* = 0.451. Conditional effects with 95% CI are displayed in the Supplementary Material. The Johnson-Neyman Technique revealed that the confidence interval was not completely above zero after an academic ability level of 6.60, which is the case for 44% of the children. Therefore, for the 56% children with lower ability levels, a more non-limited willpower theory was associated with better behavioral self-regulation than a more limited willpower theory was.

**TABLE 3:** Model coefficients of the moderation of academic ability level on the relation between willpower theories and behavioral self-regulation, controlling for age, and gender.

		Coeff.	*SE*	*t*	*P*	CI_95_ lower	CI_95_ upper
Intercept		–1.237	15.472	–0.080	0.936	–31.848	29.375
Willpower theories (WT)	*b*_1_	8.275	2.469	3.351	0.001	3.390	13.160
Academic ability level (AAL)	*b*_2_	4.573	1.127	4.058	0.000	2.343	6.802
WT × AAL	*b*_3_	–1.012	0.362	–2.793	0.006	–1.728	–0.295
Gender	*b*_4_	–3.424	1.638	–2.091	0.038	–6.664	–0.184
Age	*b*_5_	0.185	0.177	1.042	0.299	–0.166	0.536

**F*(5, 129) = 7.798, *p* = 0.000, Adjusted *R*^2^ = 0.202; *R*-square increase due to interaction: Δ*R*^2^ = 0.046, *F*(1,129) = 7.801, *p* = 0.006.*

#### Mediation Through Goal Orientation

**FIGURE 2:**
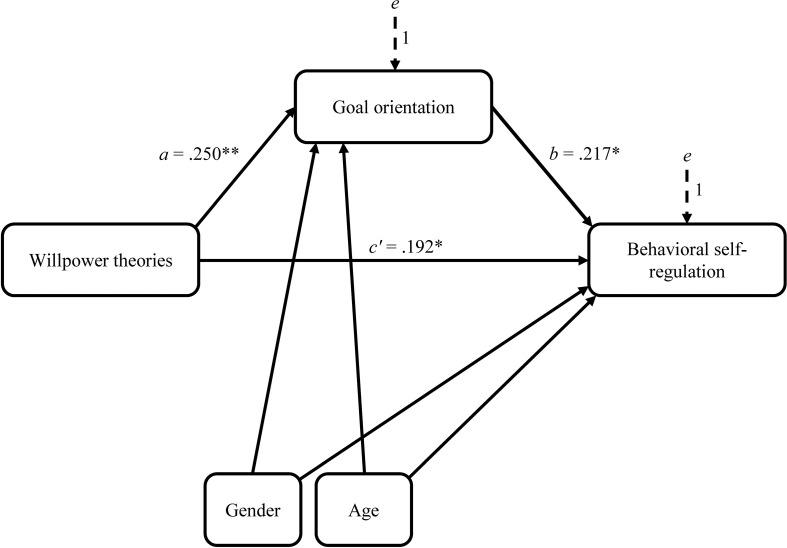
Statistical diagram of the mediation model with standardized regression coefficients for presumed influence of willpower theories on behavioral self-regulation through goal orientation with statistical controls; ^∗^*p* < 0.05, ^∗∗^*p* < 0.01.

The simple mediation analysis (Model 4 in PROCESS) conducted using ordinary least square path analysis showed that willpower theories directly and indirectly influenced behavioral self-regulation through its effect on learning goal orientation (Figure 2). As Table 4 shows, children who thought of their willpower as non-limited were more learning oriented than children with limited theories were (*a* = 0.280, *p* = 0.004, 95% CI [0.093, 0.466]), and children who were more learning oriented showed better behavioral self-regulation than children who liked to do easy tasks that they had already mastered (*b* = 1.909, *p* = 0.014, 95% CI [0.391, 3.427]). The completely standardized regression coefficients are displayed in Figure 2 and Table 4. A bootstrap confidence interval for the indirect effect (*ab* = 0.534) based on 10,000 bootstrap samples showed that this effect was statistically different from zero as revealed by the 95% bias-corrected bootstrap confidence interval entirely above zero (95% CI [0.100, 1.347]). A partially standardized indirect effect of 0.052, 95% CI [0.009, 0.124] and a completely standardized effect size of 0.053, 95% CI [0.010, 0.129]) revealed a small partial mediation with a significant ratio of indirect to total effect of willpower theories on behavioral self-regulation (0.220, 95% CI [0.038; 1.003]). The direct effect of willpower theories remained significant, indicating that they were related to behavioral self-regulation independent of their effect on learning goal orientation (*c*′ = 1.889, *p* = 0.029, 95% CI [0.190, 3.588]).

**TABLE 4:** Model coefficients and completely standardized regression coefficients for the conditional direct and indirect effects of willpower theories on behavioral self-regulation, through goal orientation.

		Goal orientation					Behavioral SR			
		β	Coeff.	*SE*	*p*		β	Coeff.	*SE*	*p*
Willpower theories	*a*	0.250	0.280	0.094	0.004	*c′*	0.192	1.889	0.859	0.029
Goal orientation		–	–	–	–	*b*	0.217	1.909	0.767	0.014
Gender		0.133	0.313	0.195	0.111		–0.200	–4.136	1.737	0.019
Age		0.068	0.017	0.021	0.412		0.080	0.178	0.185	0.338
Constant	*i*_*M*_		1.064	1.584	0.503	*i*_*Y*_		21.537	13.988	0.126
	*R*^2^ = 0.102					*R*^2^ = 0.132				
	*F*(3,132) = 4.972, *p* = 0.003					*F*(4,131) = 4.969, *p* = 0.001				

## Discussion

This study is the first to demonstrate that kindergarteners already have distinct and varying ideas about the nature of their willpower that can be assessed reliably. Children’s implicit willpower theories range from a non-limited to a limited theory in low and high achievers and are distinguishable from related concepts such as a learning goal orientation. Importantly, this study shows that kindergarteners’ beliefs about the nature of willpower are related to behavioral self-regulation. Children who agreed more that they needed a break after strenuous tasks (limited theory) performed worse in the behavioral self-regulation task than children who rather agreed that exerting willpower is energizing (non-limited theory). Further, our results support the hypothesized moderation by academic ability levels: willpower theories are especially beneficial for children with a low ability level. We also found support for the hypothesized mediation: The more children endorsed a non-limited theory about their willpower the more they expressed a preference for challenging tasks in order to learn, which accounted for their better performance in behavioral self-regulation. These results support our assumption that a limited willpower theory in children is associated with a preference for easy tasks. We assume that Children with a limited theory avoid difficult tasks so as not to strain their willpower and therefore seldom train their behavioral self-regulation.

One of the main questions leading this research was why some children come to effectively regulate their behavior, which is related to better adaption and performance in school, while others struggle. The results highlight the possibility that behavioral self-regulation may not only depend on biological predisposition or develop as a result of repeated training, as examined in past research (Walk and Evers, 2013; Diamond and Ling, 2016). It might also develop through an implicit understanding of willpower as non-limited. Haimovitz et al. (2019) proposed that “if children learn to approach willpower as self-energizing, this could develop into a more general tendency to search for strategies and be resourceful across multiple novel self-regulatory situations” (p. 7). Besides this rather direct relationship to improved behavioral self-regulation in challenging situations, our results also highlight an indirect relationship. Viewing willpower as more of a limited resource is relates to a less pronounced learning goal orientation and therefore may lead to an avoidance of challenging, strenuous tasks. In turn, opportunities to train behavioral self-regulation in the face of difficulties get lost. If children continue to avoid challenging tasks, this may become a pronounced hindrance over time, since challenge is important for training self-regulation (Diamond and Lee, 2011). A non-limited theory therefore might be especially beneficial in the early childhood years. During this time autonomous play and learning environments are more common than later in primary school, where the selection of task difficulty becomes more externally controlled by teachers than by children’s own motivational beliefs. Especially in newer adaptive teaching and learning concepts, which are based on the assumption of a self-regulated active individual, willpower theories may play an important role for task selection, strategy use, and persistence. Swiss kindergartens emphasize open learning environments, and it may be that the interaction of willpower theories, academic ability level, and behavioral self-regulation is different in more structured environments, where there is less free play and free choice. On the other hand, the greater autonomy in kindergartens might foster a non-limited willpower theory. Sieber et al. (2019) showed that autonomous goal striving promotes the endorsement of non-limited theories mediated through vitality, the experience that tasks are energizing. However, autonomous task selection and pursuit might also be challenging and overtaxing for some children (Sieber et al., 2016). The change from strong external regulation by primary caregivers in early childhood to complete internalization of regulation is a central process in the development of self-regulatory competence (Kochanska et al., 2001). Therefore, support from teachers is crucial also in autonomous settings to enable children to experience tasks as not draining but stimulating.

Future research should look at the role willpower theories play in different teaching and learning settings. Especially longitudinal designs are important, with multiple variables to assess developmental patterns after the transition from kindergarten to primary school, where the educational setting often changes dramatically.

Various previous studies have shown that the behavioral self-regulation task used in this study is predictive of achievement later in school and that it measures children’s performance in working memory, attention, and inhibition. This is what children need to successfully regulate themselves in classrooms, where they must actively remember instructions from the teacher, focus on the task at hand, and ignore distractions. Since the relation between a more non-limited willpower theory and behavioral self-regulation was especially pronounced in children with low academic ability levels, willpower theories may represent a resilience factor against poor performance. In this study, willpower theories did not seem to be related directly to behavioral self-regulation in children with high academic ability levels. We were able to rule out that the measurements for willpower theories and behavioral self-regulation were not sensitive enough in the upper ranges. But it might be that a non-limited theory has positive effects for children with high academic abilities in other areas of self-regulation, such as in the use of different or more effective learning strategies (Haimovitz et al., 2019).

In the present study we tested two distinct models (moderation by ability level and mediation through goal orientation) concerning the relation between willpower theories and self-regulation. An open question is, whether these two models can be combined within one more comprehensive model. It could be that academic ability level also influences the indirect effect between willpower theories and behavioral self-regulation, as ability level might moderate the relationship between willpower theories and goal orientation. Previous research suggests that learning goal orientations are independent from ability level (Dweck and Leggett, 1988). However, theoretical and empirical results on the topic are inconclusive. Children on low ability levels with a limited theory are possibly more prone than children on high ability levels to choose tasks they already master as their academic self-beliefs are lower (Marsh and Martin, 2011; Schloz and Dresel, 2011). On the other hand, high achieving children with a limited theory, who think that their resources become depleted might be just as interested in choosing tasks they already master to protect their higher self-concepts (Bouffard and Narciss, 2011; Butler, 2011). In order to generate first insights into that relationship, additional explorative moderated mediation analyses were conducted. However, in those analyses we found no evidence, that the indirect effect from willpower theories to behavioral self-regulation by goal orientation was moderated by academic ability level (see Supplementary Material for details on the moderated mediation, Model 7 in PROCESS).

A second possibility could be that ability level moderates the relationship between goal orientation and behavioral self-regulation. Previous research documents that interventions promoting a growth mindset, which is supposed to promote learning goal orientation, are specifically beneficial among lower achieving students (Paunesku et al., 2015; Yeager et al., 2019). Apparently, low ability students are the ones whose performance depends more heavily on their motivational orientation toward learning and effort engagement. High performing students might float through academic settings without high effort expenditure. Accordingly, we conducted a second explorative analysis (Model 14 in PROCESS). Again, we found no evidence, that the indirect effect from willpower theories to behavioral self-regulation by goal orientation was moderated by ability level (see Supplementary Material for details on the moderated mediation). Thus, future research should further investigate the relationship between willpower theories, learning goal orientation and self-regulation based on individual students’ academic ability levels and may also include self-concepts and self-efficacy as mediators.

Our results highlight that willpower theories already vary widely in kindergarteners, and Dweck (2017) puts the formation of mindsets at the center of development from birth. This raises the important question about the origins of willpower theories. From a developmental and evolutionary psychological perspective, a strong orientation toward exerting willpower, effort, and persistence after failure may be expected in all young children, who face challenges almost on a daily basis when learning to walk, talk, or ride a bike. When and how do the two different mindsets start to develop? Haimovitz et al. (2019) see the development of mindsets as a result of socialization and changeable by various environmental influences. There is hardly any research on possible influencing factors during child development. Studies on implicit intelligence theories suggest that contextual factors, such as feedback from significant others, may have an impact (Gunderson et al., 2013, 2018). Model learning certainly also plays a central role in the development of implicit theories. If children see that significant others experience challenges as energizing, a non-limited mindset may be promoted (Haimovitz et al., 2019). Conceivably, parents or teachers who display depleted energy and a need for recreation after a challenging workday may set an example for a limited theory. However, it should always be remembered that taking breaks, as a motivational strategy in the sense of a self-reward and not as cause of depleted resources, is a highly recommended self-regulatory strategy (Wolters, 2003). In a recent study, Bernecker and Becker (2020) emphasize that a balance between long-term goals (i.e., learning to read) and hedonic goals (i.e., pleasure) is paramount to adaptive self-regulation. It makes a difference whether children struggling with a task take a break because they believe their resources are depleted (“I’m exhausted”) or take a break as a reward for a job well done (“I’ve earned a break!”).

Therefore, teachers and parents may play a crucial role in the forming of willpower theories. Further, it is plausible that teachers’ approaches to instruction may lead to differences in the associations between willpower theories and self-regulation in students. Interventions should look deeper into the assumed causal relation between self-regulation and willpower theories as well as possible mechanisms that affect kindergarteners’ implicit theories. Classroom practices such as low autonomy during goal striving (Sieber et al., 2019) as well as innocuous advice from practitioners, such as “take a break after strenuous tasks,” might promote a limited willpower theory. This would have possible negative consequences for behavioral self-regulation and subsequently hinder a child’s academic development overall. As a consequence, teachers might be encouraged to be sensitive to subtle linguistic cues and to their own behavior as role models. As people with a limited theory are sensitive to the availability of mental resources (Haimovitz et al., 2019), teachers might possibly influence children’s mindsets. Future research should explore the salience and effect of different cues and instructional practices that may foster a non-limited willpower theory in the school context.

Although this study expands previous findings, there are some limitations that should be addressed. First, the correlational nature of this study precludes any claims of causation. As previous studies with students (Job et al., 2010) and preschoolers (Haimovitz et al., 2019) showed that experimentally manipulated willpower theories caused a difference in self-control or delay of gratification, for example, we believe that the presented theoretical assumptions and previous empirical findings justify the assumption of a causal process. Nevertheless, it is possible that behavioral self-regulation and willpower theories influence each other and that the development of a person’s willpower theories is partly a result of metacognitive experiences, knowledge, and skills during the self-regulation process. For example, if a child struggles with a challenging task and cannot successfully complete it, the attribution of the self-regulation failure to limited willpower that has to be replenished seems reasonable. A limited theory would therefore be the consequence of self-regulation failure and not the reason. As in the present study willpower theories and academic ability levels show a medium correlation, future intervention studies should look into academic achievement as an outcome. It might be assumed that the positive constellation of non-limited willpower theories, learning goal orientation, and behavioral self-regulation must be reflected in later achievement. Therefore, kindergarteners with low academic ability levels who adopt non-limited willpower theories and show high behavioral self-regulation may show a positive development trend of academic achievement during primary school. Non-limited willpower theories might act as a motivational precondition for positive academic development.

Second, further research should validate the newly developed instrument to assess willpower in children. The items that we developed for this study may not be feasible for other age groups (e.g., younger children might have only early forms of mindsets) and cultures, as research points out that there are differences in willpower theories across cultures (Savani and Job, 2017). As willpower theories in children in this age group had not been measured up to now, future studies could explore if the manipulation of children’s beliefs (e.g., as in the study of Haimovitz et al., 2019) only affects short-term behavioral self-regulation in the experimental situation or if it also affects underlying beliefs about willpower.

Third, we assessed academic ability levels by teacher ratings of students’ academic abilities, which has advantages and disadvantages. With no formal grades given in kindergarten, teachers’ assessment of students’ abilities are valid judgments, and the kindergarten group as social reference norm is an important indicator (Marsh et al., 2002). Social comparison processes are an important developmental process for the validation of self-perception in kindergarten. This approach leads to a small variance across kindergarten classes but represents more than a mere reflection of students’ academic abilities, because teacher ratings also take motivational characteristics into account. Future research should consider the use of both achievement tests and teacher ratings but as separate latent constructs, since they have different psychological meanings (Pinxten et al., 2010).

Further, although the assessment of learning goal orientation as children’s willingness to exert effort to learn something vs. choosing easy tasks that they already master on a unidimensional scale is acceptable for this age group (Gunderson et al., 2018; Compagnoni et al., 2019), future studies should try to capture differentiated goal orientations (e.g., performance/mastery, avoidance/approach) to fully address the correlates and relations between willpower theories, behavioral self-regulation, and goal orientation.

In sum, this study suggests that willpower theories in young children can be reliably assessed, which opens up exciting new avenues for theory and application of self-regulation research. The present research shows that kindergarteners who think that willpower is limited already self-regulate less well than their peers with a non-limited view, and they prefer to do easy tasks that they already master. This holds especially true for children with lower academic achievement levels. Early behavioral deficiencies are known to be problematic for school transitioning and future learning behavior (Blair and Raver, 2015). Therefore, research on motivational beliefs (e.g., willpower theories) in this young age group is required to better understand the processes involved in the development of self-regulation. Future research should investigate mechanisms that affect willpower theories of kindergarteners, to foster a view of their own willpower as energizing. This has the potential to promote behavioral self-regulation and possibly ensure long-term academic success.

## Data Availability Statement

The raw data supporting the conclusions of this article will be made available by the authors, without undue reservation.

## Ethics Statement

The studies involving human participants were reviewed and approved by Ethics Commitee, Faculty of Arts and Social Sciences, University of Zurich, Switzerland. Written informed consent to participate in this study was provided by the participants’ legal guardian/next of kin.

## Author Contributions

MC conceived and designed the study, developed the new measure, collected and analyzed the data, and wrote a first version of the manuscript. VS verified the analyses. VS and VJ provided advise and discussed the results. All authors wrote the final manuscript.

## Conflict of Interest

The authors declare that the research was conducted in the absence of any commercial or financial relationships that could be construed as a potential conflict of interest.
