# Supplementary material for: My Brain Needs a Break: Kindergarteners’ Willpower Theories Are Related to Behavioral Self-Regulation
Source: Front Psychol. 2020 Dec 16;11:601724. doi: 10.3389/fpsyg.2020.601724 (PMC7772190; doi:10.3389/fpsyg.2020.601724)
Supplement: Supplementary file 1 [file Data_Sheet_1.pdf]

Supplementary Material

1 Items assessing willpower theories in kindergarteners

Items included in the measure of willpower theories given to children aged 65–86 months (items adapted from Job et al. (2010); method inspired by the Berkeley Puppet Interview [Measellle et al., 1998][1]). Items marked “A” were given at the first session, and items marked “B” were given at the second session. Items were administered in a single, pseudo-random order. Items marked with an asterisk (*) were reverse-coded, so that higher scores were associated with a nonlimited theory. On a 5-point semantic differential scale displayed on the touchscreen between the two puppets, children indicated which of the puppets they could identify with (1 = limited theory, 5 = nonlimited theory).

1. A: *Wenn du fescht hesch müese denke. Wie isch das für din Chopf? 1 = Denn bechumi grad Lust zum nomol öppis schwirigs mache. 5 = Denn bruchi zerscht e Pause, bevor min Chopf wider denke chan. *English translation: If you had to think very hard. How is that for your brain?* 1 = *Then I just get the urge to do something difficult again,* 5 = *Then I need a break before my brain can think again.*

2. A: Brucht din Chopf vill Pause bim denke? 1 = Jo, immer wenni öppis astrengends gmacht ha, brucht min Chopf e Pause. 5 = Nei gar nöd, min Chopf cha so lang denke wie ich will. *English translation: Does your brain need many breaks during strenuous thinking?* 1 = *Yes, whenever I have done something strenuous, my brain needs a break,* 5 = *Not at all, my brain can think as long as it wants.*

3. A: Wenn din Chopf müed isch, chasch du denn im Chindsgi no guet zuelose?“ 1 = Nei, denn bruchi e Pause. 5 = Jo, weni will scho. *English translation: If your brain is tired, can it still listen well in kindergarten?* 1 = *No, then I need a break,* 5 = *Yes, if I want to.*

1. B: Wenn du fescht hesch müese denke. Wie isch das für din Chopf? 1 = Denn bruchi zerscht e Pause, bevor min Chopf wider denke chan. 5 = Denn bechumi grad Lust zum nomol öppis schwirigs mache. *English translation: If you had to think very hard. How is this for your brain?* 1 = *Then I need a break before my brain can think again,* 5 = *Then I just get the urge to do something difficult again.*

2. B: *Brucht din Chopf vill Pause bim denke? 1 = Nei gar nöd, min Chopf cha so lang denke wie ich will. 5 = Jo, immer wenni öppis astrengends gmacht ha, brucht min Chopf e Pause. *English translation: Does your brain need many breaks during strenuous thinking?* 1 = *Not at all, my brain can think as long as it wants,* 5 = *Yes, whenever I have done something strenuous, my brain needs a break.*

3. B: *Wenn din Chopf müed isch, chasch du denn im Chindsgi no guet zuelose?“ 1 = Jo, weni will scho. 5 = Nei, denn bruchi e Pause. *English translation: If you had to think very hard. How is this for your brain?* 1 = *Then I just get the urge to do something difficult again, 5 = Then I need a break before my brain can think again.*

[1] Many thanks to Jamie J. Jirout for her advice on scale development.

2 Supplementary Figures

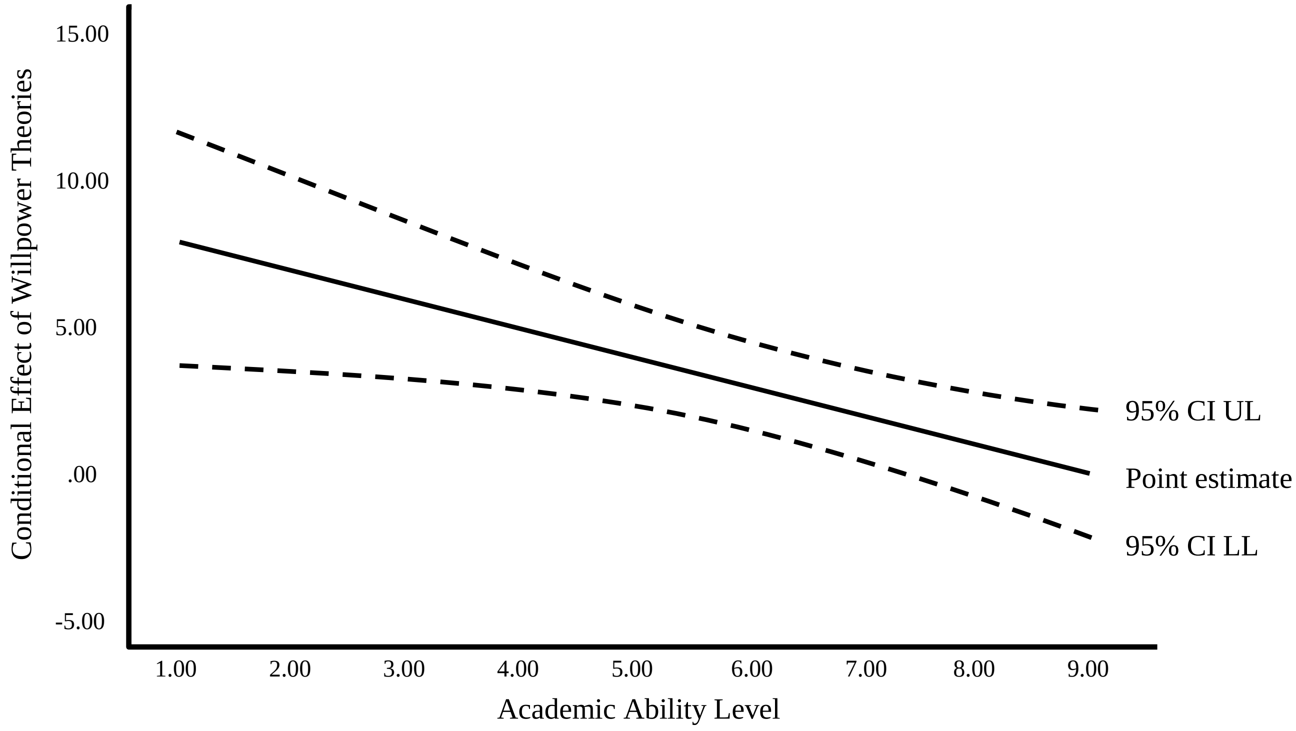

Supplementary Figure 1. Conditional effect of willpower theories on behavioral self-regulation as a function of academic ability level. Graphical depiction of the moderation using the Johnson-Neyman Technique; the solid black line is the conditional effect, and the dotted lines are the upper and lower bounds of a 95% confidence interval.

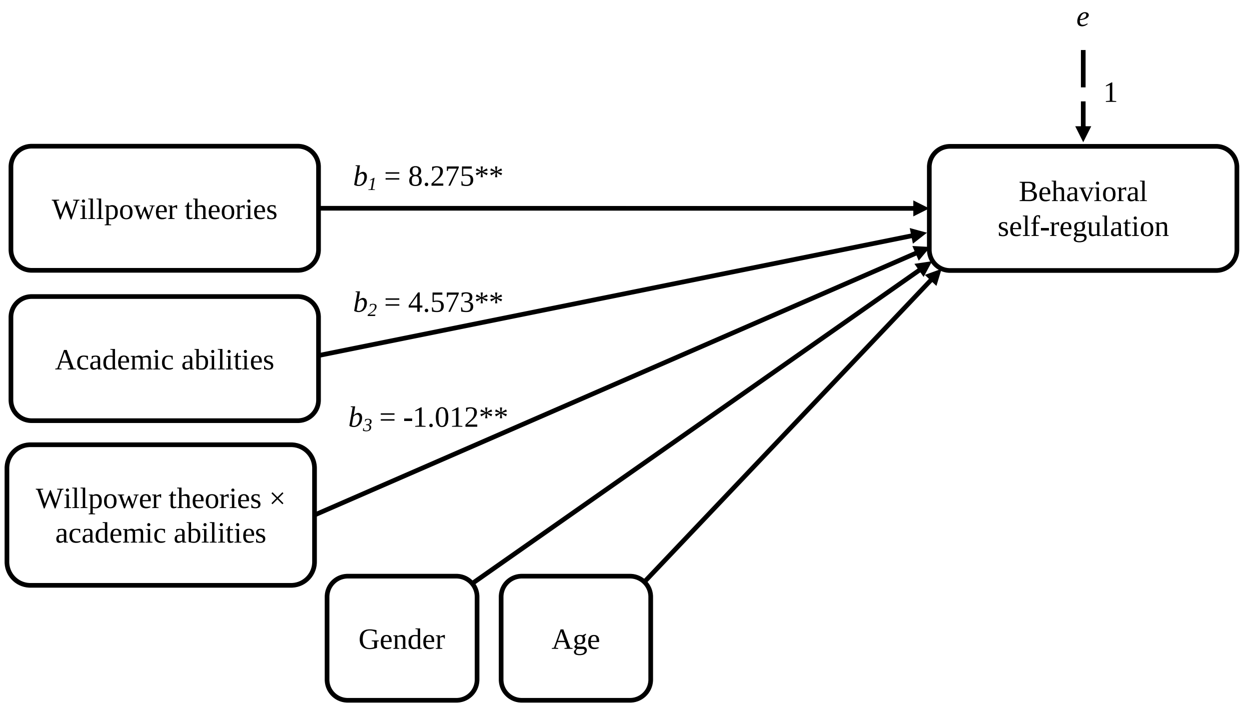

Supplementary Figure 2. Statistical diagram of the simple moderation model with academic achievement as moderator of the direct effect of willpower theories on behavioral self-regulation with statistical controls; * $p < .05$, ** $p < .01$.

3 Explorative moderated mediation analyses

We conducted two explorative moderated mediation analyses to see whether the indirect effect of willpower theories on behavioral self-regulation through goal orientation was moderated by ability level. Gender and age were included as covariates. In the first explorative moderated mediation analysis (Model 7 in PROCESS; Hayes, 2018) we tested whether ability level moderated the link between willpower theories and goal orientation in our mediation model (see supplementary figure 3). As the model coefficients show (supplementary table 1), there was no significant interaction of willpower theories and academic ability level on goal orientation (a_3 = -0.011, p = .794, 95% CI[-0.095; 0.073]) and no significant conditional indirect effect (Index of moderated mediation = -.022, 95% CI[-.290; .127]). The significant direct effect (direct effect = 1.894, p = .029, 95% CI [0.189, 3.598]) indicates that children with more nonlimited willpower theories show better behavioral self-regulation independent of the moderated indirect effect.

In the second explorative moderated mediation analysis (Model 14 in PROCESS; Hayes, 2018) we tested whether ability level moderated the link between goal orientation and behavioral self-regulation in our mediation model (see supplementary figure 4). As the model coefficients show (supplementary table 2), there was no significant interaction of goal orientation and academic ability level on behavioral self-regulation (b_3 = -0.228, p = .543, 95% CI[-0.966; 0.511]) and no significant conditional indirect effect (Index of moderated mediation = -.063, 95% CI[-0.377; 0.145]). The positive direct effect indicates that children with more nonlimited willpower theories show better behavioral self-regulation, when holding constant the indirect effect. However, this effect is not statistically significant (direct effect = 1.476, p = .082, 95% CI [-0.188, 3.140]).

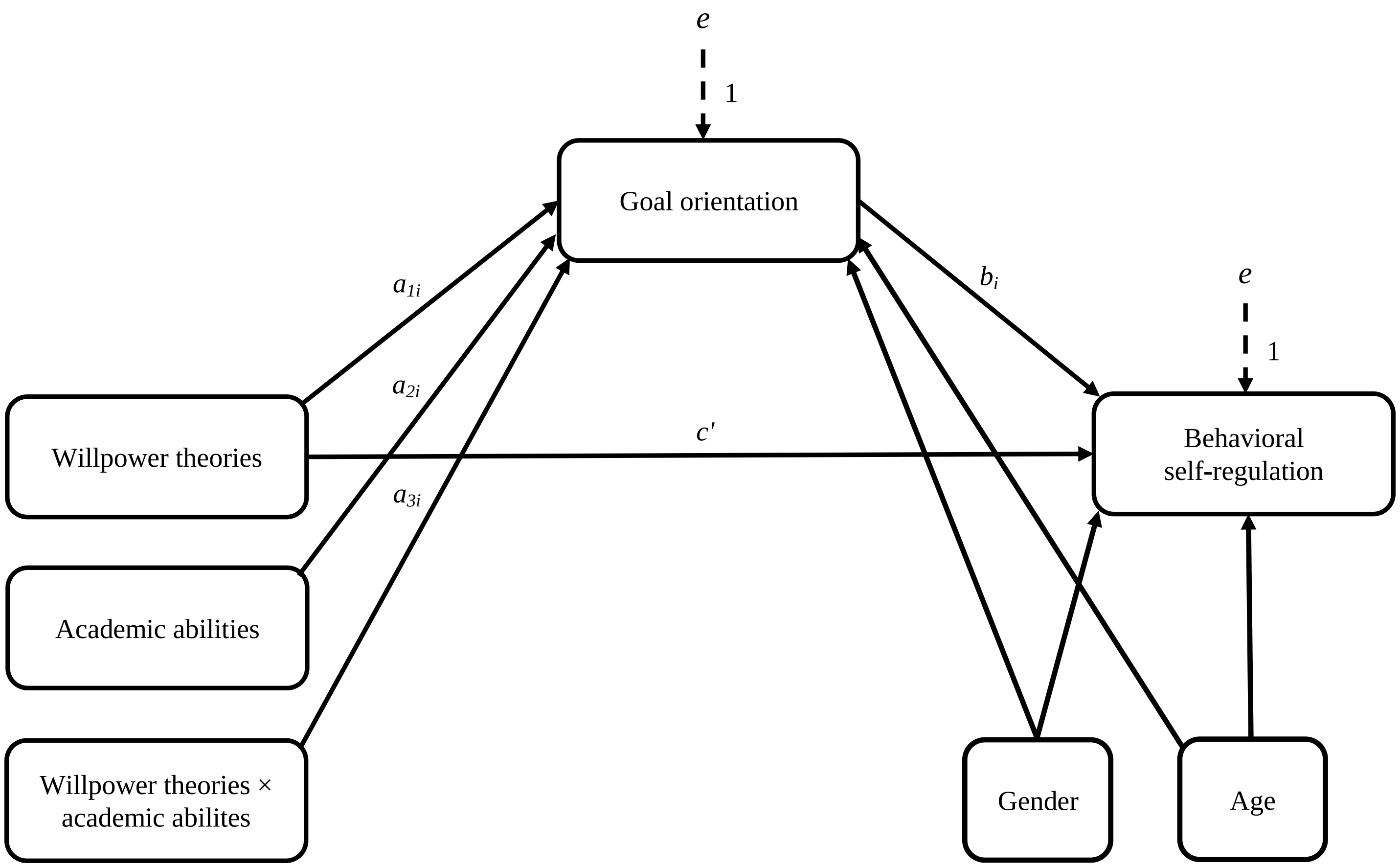

Supplementary Figure 3. Statistical diagram for the moderated mediation model (Model 7 in PROCESS) with a conditional indirect Effect (a_{1i} + a_{3i} × AAL)b_i) of willpower theories on behavioral self-regulation through goal orientation.

Supplementary Table 1

Model coefficients for the moderated mediation model (Model 7 in PROCESS) with a conditional indirect Effect (a_{1i} + a_{3i} × AAL)b_i) of willpower theories on behavioral self-regulation through goal orientation

		Goal orientation				Behavioral SR		
		Coeff.	*SE*	*p*		Coeff.	*SE*	*p*
Willpower theories	a_1	0.297	0.290	.308	c'	1.894	0.861	.029
Goal orientation		-	-	-	b	1.944	0.774	.013
Ability level (AAL)	a_2	0.155	0.132	.244		-	-	-
AAL × WT	a_3	-0.011	0.043	.794		-	-	-
Gender		0.307	0.192	.114		-4.078	1.747	.021
Age		0.014	0.021	.492		0.170	0.186	.363
Constant		0.499	1.819	.784		21.883	14.052	
		R^2 = .143<br>F(5,129) = 4.322, p = .001				R^2 = .132<br>F(4,130) = 4.959, p = .001		

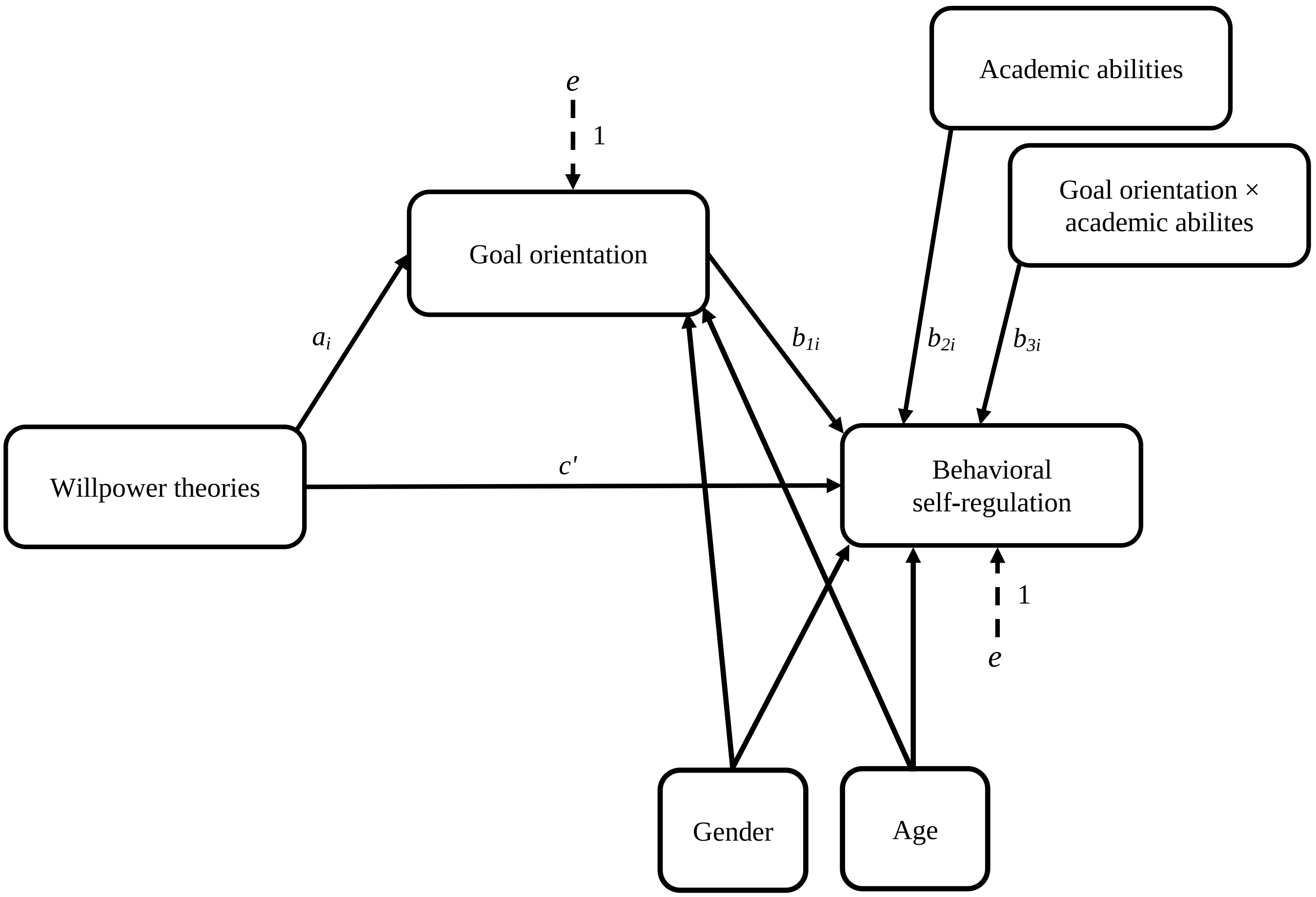

Supplementary Figure 4. Statistical diagram of the moderated mediation model (Model 14 in PROCESS) with a conditional indirect Effect ($a_i(b_{1i} + b_{3i} \times AAL)$) of willpower theories on behavioral self-regulation through goal orientation.

Supplementary Table 2

Model coefficients for the moderated mediation model (Model 14 in PROCESS) with a conditional indirect Effect ($a_i(b_{1i} + b_{3i} \times AAL)$) of willpower theories on behavioral self-regulation through goal orientation

		Goal orientation				Behavioral SR		
		Coeff.	*SE*	*p*		Coeff.	*SE*	*p*
Willpower theories	a_1	0.275	0.094	.004	c'	1.476	0.841	.082
Goal orientation (GO)		-	-	-	b_{1i}	2.680	2.281	.242
Ability level (AAL)		-	-	-	b_{2i}	2.328	1.468	.115
AAL × GO		-	-	-	b_{3i}	-0.228	0.373	.543
Gender		0.292	0.196	.138		-3.603	1.697	.036
Age		0.019	0.021	.357		0.112	0.180	.536
Constant		0.950	1.585	.550		15.094	15.718	.339
		$R^2 = .100$<br>$F(3,131) = 4.844$, $p = .003$				$R^2 = .208$<br>$F(6, 128) = 5.598$, $p = .000$		
